# Fatores Preditores de Risco de Sangramento em Pacientes Submetidos a Cirurgia Valvar

**DOI:** 10.36660/abc.20230453

**Published:** 2024-10-08

**Authors:** Alef de Carvalho Vieira, Renato Tambellini Arnoni, Ana Beatriz Silva Barbosa, Attila Santos Berriel, Rafael Guimarães Vianna, Mario Issa

**Affiliations:** 1 Instituto Dante Pazzanese de Cardiologia São Paulo SP Brasil Instituto Dante Pazzanese de Cardiologia, São Paulo, SP – Brasil; 2 Hospital Edmundo Vasconcelos São Paulo SP Brasil Hospital Edmundo Vasconcelos, São Paulo, SP – Brasil

**Keywords:** Valvopatia Aórtica, Procedimentos Cirúrgicos Cardiovasculares, Complicações Pós-Operatórias, Fatores de Risco

## Abstract

**Fundamento:**

O pós-operatório de cirurgia cardíaca valvar é desafiador devido ao risco de sangramento, levando a complicações e aumento da morbimortalidade.

**Objetivo:**

Desenvolver um escore de risco para prever hemorragia em pacientes no pós-operatório de cirurgia valvar.

**Métodos:**

Estudo retrospectivo de pacientes submetidos a cirurgia valvar entre 2021 e 2022 no IDPC. Pacientes com sangramento maior foram selecionados com base nos critérios de BARC e Bojar. Foi realizada uma análise de regressão logística para fatores relacionados ao sangramento e foi criado um nomograma. Para significância estatística, foram considerados p<0,05 e um intervalo de confiança de 95%. O estudo foi aprovado pelo CEP.

**Resultados:**

Foram analisados 525 pacientes com idade média de 56 anos e predomínio do sexo feminino. A valvopatia mais comum foi a insuficiência mitral, 8,8% apresentaram sangramento aumentado e houve 4,3% de reabordagens cirúrgicas. As variáveis com significância estatística foram: insuficiência tricúspide (OR 3,31, p < 0,001), doença renal crônica/lesão renal aguda (OR 2,97, p = 0,006), hemoglobina pré-operatória (OR 0,73, p < 0,001), reoperações (OR 2,5, p = 0,003), tempo de circulação extracorpórea (CEC) (OR 1,12, p < 0,001), abordagem de 2 valvas OR de 2,23 (p = 0,013), uso de concentrado de hemácias OR de 2,8 (p = 0,001). No modelo múltiplo a insuficiência tricúspide, tempo de CEC e hemoglobina pré-operatória alcançaram significância estatística.

**Conclusão:**

O tempo de CEC, hemoglobina pré-operatória e insuficiência tricúspide associaram-se independentemente com hemorragia pós-operatória. A escala proposta é plausível, e pode auxiliar na predição de risco de sangramento.

## Graphical abstract

**Figure:**
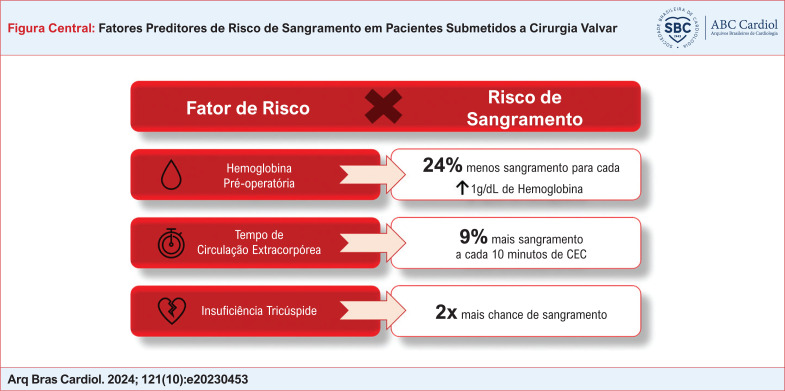

## Introdução

As doenças cardiovasculares (DCVs) representam uma das principais causas de morte em todo o mundo. O envelhecimento da população, a obesidade, o sedentarismo e as doenças crônicas contribuem para o aumento da prevalência das DCVs.^1,2^

O coração humano possui quatro válvulas: mitral, aórtica, tricúspide e pulmonar. As doenças que afetam essas estruturas representam uma parcela significativa das DCVs e são responsáveis por cerca de um terço das cirurgias cardíacas realizadas no Brasil.^3^

O tratamento cirúrgico das valvopatias apresenta desafios, e um dos mais complexos é o manejo pós-operatório. Nessa fase, surgem inúmeras complicações, e o sangramento segue sendo uma das principais, ocorrendo em aproximadamente 20% dos casos.^4,5^ A utilização da circulação extracorpórea (CEC),^6–9^ o estresse cirúrgico, a hipotermia, a resposta inflamatória e as lesões de isquemia-reperfusão são fatores que contribuem para alteração da cascata de coagulação.^6,10^

As reoperações enfrentam um risco ainda maior de complicações pois é necessário realizar descolamento de aderências, lidar com tecidos friáveis e prolongar o tempo de CEC. Esses fatores combinados contribuem para o aumento do sangramento durante o período perioperatório.^4,8,10,11^

**Figura 1:**
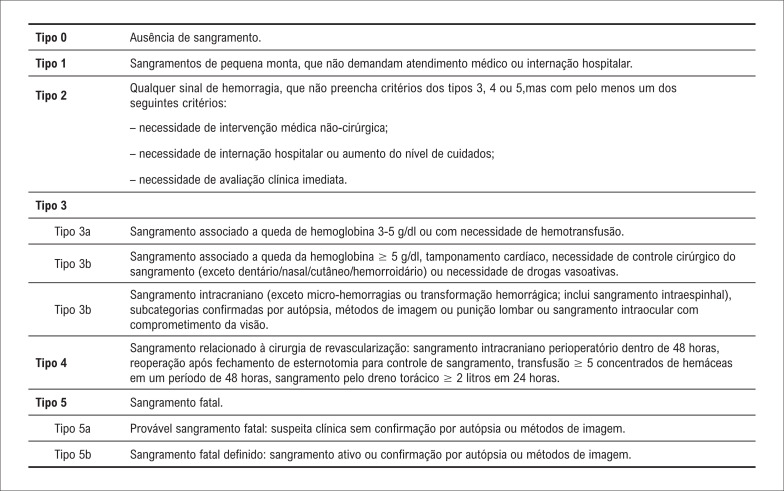
Classificação do *Bleeding Academic Reasearch Consortium* (BARC).^13^

O critério para definir sangramento aumentado no pós-operatório varia entre os autores.^5,12^ O Bleeding Academic Research Consortium (BARC) estabelece critérios específicos para classificar o sangramento, variando de 0 a 5, onde o grau 0 indica ausência de sangramento e o grau 5 representa um sangramento fatal (Figura 1).^13^ Outros critérios mais detalhados são apresentados por Bojar^14^ que avalia o sangramento a cada hora nas primeiras horas do pós-operatório (Tabela 1). Kouchoukos et al.,^15^ por sua vez, fornecem indicações para o retorno ao centro cirúrgico e reoperação do paciente, levando em consideração o volume de sangue drenado por hora e a ocorrência de drenagem súbita e maciça de sangue.^4^

**Tabela 1:** Diagnóstico clínico de sangramento importante segundo Bojar^14^

**1.**	1,5 ml/kg/h por seis horas consecutivas (ou > 100-150ml/h);
**2.**	2,0 ml/kg/h por três horas consecutivas (ou > 150-200 ml/h);
**3.**	3,0 ml/kg/h por duas horas consecutivas (ou > 250-300 ml/h).

Adaptado de Bojar.^14^

A administração de hemoderivados é uma das abordagens de tratamento adotadas. Estima-se que aproximadamente 20% de todos os produtos sanguíneos sejam transfundidos no centro cirúrgico.^16^ A transfusão sanguínea pode acarretar danos ao paciente,^8,9,11,16–18^ incluindo o aumento da incidência de infecções, lesões pulmonares, isquemia, disfunção de múltiplos órgãos e um aumento global na morbi-mortalidade.^19–22^ O objetivo desse estudo foi desenvolver um escore de risco para identificar pacientes com maior probabilidade de sangramento aumentado no pós-operatório de cirurgia valvar e avaliar os fatores de risco pré, intra e pós-operatórios para sangramento.

## Métodos

### Desenho do estudo

Trata-se de um estudo de corte transversal, retrospectivo, analítico dos pacientes valvares que foram submetidos à cirurgia em um instituto de referência em cardiologia, tendo o intuito de avaliar fatores de risco para sangramento no pós-operatório desta população.

O estudo foi realizado avaliando os pacientes operados no período de janeiro de 2021 a dezembro de 2022. O pós-operatório imediato foi considerado as primeiras 12 horas após a entrada na UTI e precoce o período até 48 horas.

### Critério de inclusão e exclusão

Os critérios de inclusão foram: paciente com doença valvar adquiridas ou congênitas; Reoperações; Combinações de procedimentos envolvendo mais de uma válvula; Pacientes ≥18 anos.

Os critérios de exclusão foram: cirurgia valvar combinada com revascularização do miocárdio ou aorta; Pacientes com dados incompletos no banco de dados; Pacientes que se negaram a assinar TCLE ou que não estavam no banco de dados.

### Variáveis

De acordo com a literatura deste trabalho, foram definidas variáveis como preditores de risco para sangramento aumentado. Foram variáveis pré-operatórias: idade, peso, altura, área de superfície corpórea, gênero, reoperação, hipertensão arterial sistêmica (HAS), Coagulopatia e trombocitopenia, diabetes *mellitus*, doença renal crônica ou lesão renal aguda, uso de anti-agregante plaquetário e anticoagulante, valor de hemoglobina sérico, valor de creatinina sérico. Variáveis intraoperatórias: tempo de cirurgia, tempo de CEC, tempo de anoxia, temperatura em CEC, tipo de cirurgia realizada e associações, dose heparina, uso ou não de concentrado de hemácias e hemocomponentes e a sua quantidade, tempo de coagulação ativado pós reversão com Sulfato de Protamina e uso de sistema de autotransfusão. Variáveis pós-operatórias: Reoperação para revisão de hemostasia, quantificação de volume de sangue drenado por horário nas primeiras 6 horas, somatório das 12, 24 e 48 horas; presença de sangramento intracraniano pós-operatório, uso de concentrado de hemácias e hemocomponentes e sua quantidade e uso de medicações antifibrinolíticas, presença de acidose metabólica, óbito e óbito por sangramento.

### Extração de dados

A tabulação dos dados foi feita de maneira organizada no *software* do RedCap (*Reasearch Eletronic Data Capture)*^23^ a partir do banco de dados do setor de Valvopatias do instituto. A partir dos dados de fatores de risco foi feita a análise com a finalidade de encontrar os que mais se associam ao desfecho sangramento aumentado no pós-operatório. Para diferenciação dos grupos entre aqueles que tinham ou não sangramento aumentado, foram utilizados os critérios do BARC^13^ e Bojar.^14^ Na classificação do BARC^13^ foram utilizado os critérios do item 1 para ausência de sangramento e item 4 da Figura 1: Reoperação para revisão de hemostasia, sangramento intracraniano nas primeiras 48 horas, uso de 5 ou mais concentrados de hemácias em 48 horas, e drenagem de 2000ml de sangue nas primeiras 24 horas. Na classificação de Bojar^14^ foram avaliadoa os pacientes de acordo com o volume de sangue drenado a cada horário de pós-operatório conforme Tabela 1.

### Análise estatística

A descrição da amostra coletada foi realizada a partir de medidas de frequência absoluta e relativa para as variáveis categóricas e apresentadas a partir de medidas de posição (média, mediana) e dispersão (desvio padrão e intervalos interquartis) para as variáveis contínuas. Os efeitos das variáveis demográficas, intraoperatórias e cirúrgicas no sangramento maior foram analisados por meio de modelos de regressão logística. Inicialmente, foram realizadas análises univariadas. As variáveis com significância estatística foram incluídas no modelo múltiplo, considerando-se a colinearidade entre as variáveis, a significância estatística e a plausibilidade para entrada no modelo. O nível de significância estatística foi considerado p < 5% e intervalo de confiança de 95%.

Com base no modelo final, foi elaborado um nomograma utilizando o software R. Esse nomograma apresenta uma série de escalas com as características dos pacientes (variáveis incluídas no modelo múltiplo). Cada escala atribui uma pontuação, que, quando somada, fornece uma pontuação final para o paciente. Essa pontuação final é utilizada para avaliar o risco de sangramento do paciente.

Validações externas (em outra base de dados) não estão previstas para esse trabalho. As análises foram realizadas com o software R, versão 4.2.1.

### Questões éticas

O projeto foi aprovado pelo Comitê de Ética em Pesquisa do Instituto Dante Pazzanese de Cardiologia. Número do CAAE: 59774422.9.0000.5462. Número do Parecer: 5.497.323. Número do CEP: 5268. O TCLE foi dispensado pelo comitê.

## Resultados

### Descrição da amostra

De 590 pacientes operados por valvopatias, foram excluídos 65, e incluídos 525 pacientes na análise. A idade média foi de 56 anos, e 57,3% eram do sexo feminino, com peso médio de 73 kg, e ASC de 1,82 m². A patologia mais frequente foi a insuficiência mitral (51,2%), seguida da estenose mitral (37,7%) e estenose aórtica (33,9%), e insuficiência tricúspide com 19,6% dos casos. Das operações realizadas, 30,5% foram reoperações.

Cerca de 51% tinham HAS, 16% eram diabéticos, 9,7% tinham doença renal crônica ou lesão renal aguda e 18,7% apresentavam trombocitopenia prévia. 36,4% dos pacientes não apresentavam nenhum histórico médico prévio relevante. A média de creatinina foi de 1,11 g/dL, hemoglobina de 12,87 g/dL, tempo de tromboplastina parcial ativada de 28,04 segundos e fibrinogênio de 352 mg/dL. Dos pacientes, 5,5% usavam anticoagulantes orais não dependentes de vitamina K e 24,6% usavam varfarina no período pré-operatório.

O tempo médio de cirurgia foi de 309,5 minutos, de CEC 123,8 minutos, e de anoxia 86,91 minutos. A abordagem da valva mitral foi realizada em 67,4% dos pacientes, da valva aórtica em 43,9%, da valva tricúspide em 15,3% e da valva pulmonar em 0,6%. Em 23,4% das cirurgias, mais de uma valva foi abordada. Durante a cirurgia, 42,3% dos pacientes receberam concentrado de hemácias, com uma média de 1,94 bolsas utilizadas. Cerca de 31,6% dos pacientes receberam algum tipo de hemocomponente e, do total, 33,3% dos pacientes utilizaram uma máquina de autotransfusão.

Apenas 0,2% dos pacientes apresentaram hemorragia intracraniana nas primeiras 48 horas após a cirurgia, e 4,8% dos pacientes necessitaram de uma nova abordagem para controle do sangramento, sendo a causa mais comum o sangramento torácico de origem cirúrgica. Os pacientes apresentaram um volume de sangramento acumulado uma média de 257 ml em 12 horas, 380 ml em 24 horas e 475 ml em 48 horas. Cerca de 11,8% necessitaram de concentrados de hemácias no pós-operatório, média de 2,56 bolsas utilizadas. Houve um total de 8,8% de óbitos, sendo que 10,9% desses foram causados por sangramento.

### Variáveis relacionadas a sangramento aumentado

Na definição de BARC foi identificada uma prevalência de 5,3% (n=28) de casos de sangramento maior, e na de Bojar de 7,6% (n = 40). Ambos os critérios concordaram em 22 dos casos positivos identificados. A prevalência geral de acordo com os dois critérios foi de 8,8% (n=46). Destes, a classificação de BARC foi responsável por 61% e da de Bojar por 87% desses casos. A Tabela 2 resume as variáveis de acordo com a presença de sangramento maior.

**Tabela 2:** Variáveis relacionadas ao sangramento maior

Variáveis		Sangramento Maior	
		Não, N = 479	Sim, N = 46
**BARC**			
	Sem sangramento Maior	479 / 479 (100%)	18 / 46 (39%)
	Sangramento Maior	0 / 479 (0%)	28 / 46 (61%)
**Bojar**			
	Sem sangramento Maior	479 / 479 (100%)	6 / 46 (13%)
	Sangramento Maior	0 / 479 (0%)	40 / 46 (87%)
**Volume Sangramento 1ª Hora**		25 (0, 50)	212 (100, 344)
**Volume Sangramento 48 horas**		400 (250, 525)	1,200 (750, 1,475)
**Sangramento (BARC)**			
	Sem sangramento significativo	479 / 479 (100%)	18 / 46 (39%)
	Tipo 4	0 / 479 (0%)	1 / 46 (2,2%)
	Tipo 4a	0 / 479 (0%)	1 / 46 (2,2%)
	Tipo 4b	0 / 479 (0%)	24 / 46 (52%)
	Tipo 4c	0 / 479 (0%)	1 / 46 (2,2%)
	Tipo 5b	0 / 479 (0%)	1 / 46 (2,2%)
**Sangramento intracraniano (até 48h)**			
	Não	479 / 479 (100%)	45 / 46 (98%)
	Sim	0 / 479 (0%)	1 / 46 (2,2%)
**Nova Abordagem Cirúrgica**			
	Não	479 / 479 (100%)	22 / 46 (48%)
	Sim	0 / 479 (0,0%)	24 / 46 (52%)
**CH (até 48h)**			
	Não	447 / 479 (93%)	16 / 46 (35%)
	Sim	32 / 479 (6,7%)	30 / 46 (65%)
**Hemocomponentes (até 48h)**			
	Não	470 / 479 (98%)	16 / 46 (35%)
	Sim	9 / 479 (1,9%)	30 / 46 (65%)
**Quantidade de Concentrado de Plaquetas**		6,0 (5,0, 7,8)	8,0 (6,0, 10,0)
**Acidose Metabólica Pós-operatória Imediata**			
	Não	304 / 479 (63%)	22 / 46 (48%)
	Sim	175 / 479 (37%)	24 / 46 (52%)
**Óbito**			
	Não	447 / 479 (93%)	32 / 46 (70%)
	Sim	32 / 479 (6,7%)	14 / 46 (30%)
**Óbito por sangramento**			
	Não	32 / 32 (100%)	9 / 14 (64%)
	Sim	0 / 32 (0%)	5 / 14 (36%)

CH: Concentrado de Hemácias.

### Análise univariada de acordo com o período perioperatório

Das variáveis pré-operatórias analisadas, aquelas que alcançaram significância estatística foram insuficiência tricúspide com OR de 3,31 (IC 1,74 - 6,2, p < 0,001), presença de doença renal crônica ou lesão renal aguda com OR 2,97 (IC 1,31 - 6,23, p = 0,006), hemoglobina pré-operatória com OR 0,73 (IC 0,62 - 0,85, p < 0,001) e reoperação com OR de 2,5 (IC 1,35 - 4,62, p = 0,003).

As variáveis cirúrgicas observadas foram tempos de cirurgia mediana de 327,5 minutos, com OR de 1,05 (IC 1,03 - 1,10, p < 0,001), de CEC 130 minutos, com OR de 1,12 (IC 1,06 - 1,18, p < 0,001) e de anoxia de 90 minutos, com OR de 1,13 (IC 1,05 - 1,21, p < 0,001). Os efeitos foram avaliados considerando incrementos de 10 minutos em cada variável. Isso significa que, por exemplo, a cada aumento de 10 minutos no tempo de CEC, há um aumento de 12% na chance de sangramento maior.

Na associação da combinação de válvulas abordadas durante a cirurgia observou-se que duas válvulas estão associadas a um maior risco de sangramento significativo, com OR de 2,34 (IC 1,17 - 4,53, p = 0,013) e três válvulas com OR de 3,7 (IC 1,00 - 11,1, p = 0,028). Além disso, diferentes combinações de valvas também apresentaram associações relevantes, como a troca da válvula mitral com plastia tricúspide OR 3,4 (IC 1,33 - 8,66, p = 0,01) e a abordagem das valvas aórtica, mitral e tricúspide OR 3,79 (IC 0,96 - 12,8, p = 0,04).

O uso de concentrado de hemácias, tem associação significativa com o sangramento maior, com OR de 2,8 (IC 1,51 - 5,4, p = 0,001), e, o uso de duas ou mais unidades teve maior risco de sangramento, com OR de 1,52 (IC 1,06 - 2,16, p = 0,021). A autotransfusão foi associada a uma maior taxa de sangramento, com OR de 2,61 (IC 1,42 - 4,85, p = 0,002).

### Análise multivariada

As únicas variáveis que mantiveram algum grau de significância no modelo multivariado foram a hemoglobina pré-operatória, o tempo de CEC e a presença de insuficiência tricúspide conforme a Tabela 3. As Tabelas 4 e 5 foram destacadas avaliando-se variáveis importantes como doença renal crônica, reoperações e uso de concentrado de hemácias. Apesar de importantes, elas não alcançaram significância estatística na análise múltipla.

**Tabela 3:** Modelo de análise multivariada considerando variáveis de hemoglobina, tempo de CEC e insuficiência tricúspide, medidas de efeito (OR), IC e medidas de p

Variáveis		OR	IC 95%	Valor de p
Hemoglobina Pré-operatória		0,76	0,64; 0,89	0,001
Tempo de CEC (a cada 10 minutos)		1,09	1,03; 1,15	0,004
**Insuficiência tricúspide**				
	Não	—	—	
	Sim	2,04	1,01; 4,06	0,043

CEC: circulação extracorpórea; IC: intervalo de confiança.

**Tabela 4:** Modelo de análise multivariada considerando variáveis de hemoglobina, tempo de CEC e insuficiência tricúspide, reoperação e concentrado de hemácias, medidas de efeito (OR), IC e medidas de p

Variáveis		OR^1^	IC 95%	Valor de p
**CH intraoperatório**				
	Não	—	—	
	Sim	1,17	0,54, 2,57	0,7
**Reoperação**				
	Não	—	—	
	Sim	1,34	0,65, 2,72	0,4
Hemoglobina Pré-operatório		0,79	0,65, 0,95	0,012
Tempo de CEC/10		1,08	1,01, 1,14	0,016
**Insuficiência da Tricúspide**				
	Não	—	—	
	Sim	2,01	0,99, 3,99	0,049

CH: concentrado de hemácias; CEC: circulação extracorpórea.

**Tabela 5:** Modelo de análise multivariada considerando variáveis de hemoglobina, DRC/LRA, tempo de CEC e reoperação, medidas de efeito (OR), IC e medidas de p

Variáveis		OR	IC 95%	Valor de p
Hemoglobina pré-operatória		0,79	0,66, 0,94	0,009
Tempo de CEC/10		1,09	1,03, 1,16	0,003
**Antecedente de DRC/IRA**				
	Não	—	—	
	Sim	2,14	0,86, 4,97	0,087
**Reoperação**				
	Não	—	—	
	Sim	1,54	0,75, 3,11	0,2

CEC: circulação extracorpórea.

**Figura 2:**
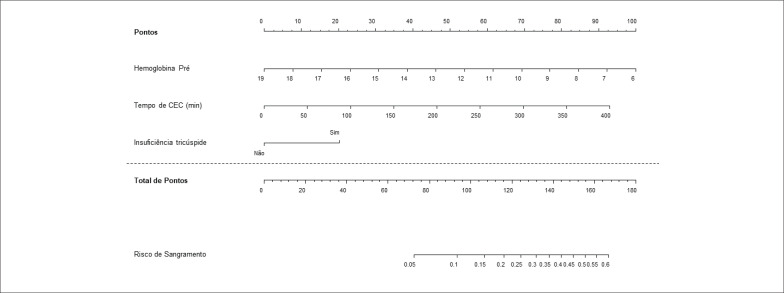
Nomograma para risco de Sangramento para modelo apresentado na Tabela 3.

Na Figura 2 e 3 encontram-se os nomogramas referentes às análises das Tabelas 3 e 5, os quais trazem consigo pontuações para determinar o risco de sangramento pós-operatório.

**Figura 3:**
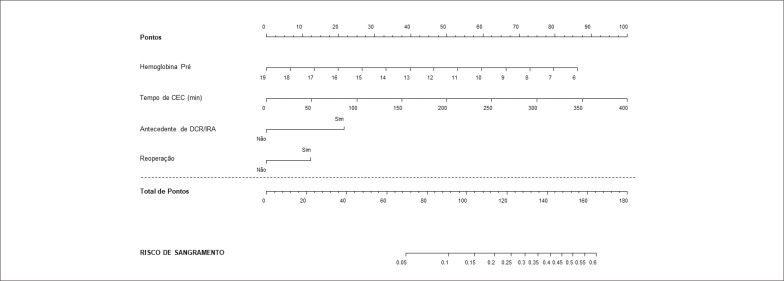
Nomograma para risco de Sangramento para modelo apresentado na Tabela 5.

## Discussão

O sangramento clinicamente aumentado apresenta variações em sua definição, o que torna sua identificação e manejo complexos. Tem causas multifatoriais e implicações no pós-operatório levando a um aumento na morbidade e mortalidade.^4,24,25^

Este estudo visou destacar as características dos pacientes submetidos a cirurgia valvar e analisar sua relação com o sangramento aumentado no pós-operatório. Observou-se que os pacientes tinham uma média de 56 anos e 57,3% eram do sexo feminino. No entanto, apesar destes dados terem relação com estudos prévios, é importante enfatizar a importância da idade acima de 75 anos, o IMC abaixo de 25 e a baixa superfície corporal como risco de sangramento.^4,26–29^

A insuficiência mitral foi identificada como a valvopatia mais prevalente neste estudo (51,2%). No entanto, em estudos anteriores, como o de Vuylsteke et al.,^29^ destacaram uma relação significativa entre a estenose aórtica e o risco de sangramento, onde, observou-se que a estenose aórtica está associada à quebra mecânica de elementos figurados e do fator de Von Willebrand, o que pode resultar em mais hemorragia. Além disso, ressalta-se que a doença mitral está frequentemente associada à presença de fibrilação atrial e ao uso de anticoagulantes cumarínicos, fatores que podem estar atrelados ao sangramento pós-operatório.^29^

Aproximadamente 8,8% dos pacientes apresentaram sangramento aumentado, com uma média de drenagem de 380 ml em 24 horas e 475 ml em 48 horas, e 4,3% precisaram realizar nova intervenção para revisão da hemostasia. Estes dados estão em consonância com estudos anteriores, o qual variou a taxa variou 6,4% a 12%, e de 2,4% e 9,5% para sangramento e reabordagens. No entanto, o volume de sangramento foi maior em outros estudos descrevendo drenagens entre 525ml e 1669ml em 24 horas.^4,10,12,24–26,28,29^

De acordo com Vuylsteke et al.,^29^ as classificações que consideram a contagem do sangramento por hora e por peso são mais confiáveis. Porém, a contabilização geral pode ser falha, porque o sangramento intraoperatório não é considerado, além das possíveis falhas humanas no momento da notificação, tornando a estimativa do volume imprecisa. Além disso, usar o número de concentrado de hemácias como marcador para definir o sangramento aumentado pode não ser preciso, pois a decisão de transfundir depende do operador.^29^

Pacientes com doença renal crônica ou lesão renal aguda apresentam piores desfechos de mortalidade em pacientes com doenças valvares.^30^ E, neste estudo viu-se a associação com sangramento aumentado, a que pode ser atribuída à redução na quantidade e qualidade das plaquetas, diminuição dos fatores de coagulação e à anemia crônica que contribui para a disfunção da coagulação.^30^

Houve uma associação positiva entre os níveis de hemoglobina pré-operatória e o sangramento pós-operatório. Os resultados deste estudo são consistentes com a média relatada por Bailly et al.,^28^ que foi de 13,6 g/dL. De acordo com os resultados deste trabalho, verificou-se que a cada aumento de 1 ponto nos níveis de hemoglobina, houve uma redução de 27% no risco de sangramento. Isso pode ser atribuído ao fato de que valores mais elevados de hemoglobina estão relacionados a um melhor transporte de substratos energéticos, contribuindo para um funcionamento adequado das plaquetas e da cascata de coagulação, além de uma menor probabilidade de necessidade de transfusão de concentrados de hemácias durante a cirurgia.

A transfusão sanguínea pode ser necessária em até 21% dos casos durante o procedimento cirúrgico e em até 5% no período pós-operatório^28^ contra 42,3% e 2,56% no presente trabalho. Além disso, o uso de duas ou mais unidades de sangue aumentou em 1,52 vezes a chance de sangramento. Essa descoberta é relevante, uma vez que a transfusão excessiva de hemácias está associada a maior mortalidade e morbidade no pós-operatório.

Observou-se que o sangramento pós-operatório em pacientes reoperados é cerca de 2,5 vezes maior, o que é concordante com outros estudos na área. Esses pacientes geralmente têm tempos de cirurgia e CEC mais prolongados devido a técnicas cirúrgicas mais delicadas e complexas necessárias durante a intervenção.^26^

O tempo de CEC está intimamente relacionado a maiores taxas de sangramento pós-operatório. O tempo de CEC desempenha um papel crucial, influenciando outros aspectos temporais, como o de cirurgia e anoxia e fatores adicionais que contribuem para um aumento na ocorrência de hemorragias. Os tempos prolongados consomem mais fatores de coagulação, resultam em danos celulares, maior fibrinólise e hemodiluição, prejudicando a capacidade de hemostasia.^4,25^ Um tempo de CEC acima de 150 minutos tem um impacto significativo no sangramento pós-operatório. Neste estudo, a cada acréscimo de 10 minutos nesse tempo, há um aumento de 12% no risco de sangramento.^4,26,30^

Há ainda um aumento no risco de sangramento à medida que mais válvulas são abordadas para troca ou plastia, podendo chegar a um aumento de 2 a quase 3,7 vezes. Essa associação é claramente influenciada pelo tempo de CEC, uma vez que é necessário mais tempo para abordar cada uma das válvulas.^26,29,30^

A insuficiência tricúspide, o tempo de CEC e a hemoglobina pré-operatória foram os principais fatores associados ao sangramento pela análise múltipla. A presença de insuficiência tricúspide como fator de risco independente difere de outros estudos que identificaram a estenose aórtica como a principal valvopatia associada ao sangramento. A razão para essa discrepância ainda não está claramente estabelecida, mas pode estar relacionada à incidência da insuficiência tricúspide e às diferentes abordagens cirúrgicas adotadas quando há outras valvopatias envolvidas.^4,12,24,26,29^

Variáveis como uso de concentrado de hemácias, reoperações e doença renal crônica desempenham um papel importante na determinação da taxa de sangramento pós-operatório, embora não de forma independente. Além disso, a presença de doença renal crônica é conhecida por sua fisiopatologia que contribui para o aumento do risco de hemorragias, mas não foi observada uma associação independente neste estudo, nem foram considerados possíveis fatores confundidores.^30^

Outros autores também destacaram diferentes fatores de risco importantes que devem ser levados em consideração como idade avançada, cirurgias não eletivas e a dose de heparina/protamina utilizada durante o procedimento cirúrgico.^4,12,26,29^

Dessa forma a criação do nomograma pode ser útil para estimar o risco de sangramentos dos pacientes. No entanto, é necessário realizar a validação externa desse escore por meio de outros estudos na área.

## Conclusão

Com base nas análises realizadas, conclui-se que o tempo de CEC, a presença de insuficiência tricúspide e a hemoglobina pré-operatória estão independentemente associados ao sangramento pós-operatório.

Outras variáveis também se mostraram importantes na associação com o sangramento pós-operatório, incluindo doença renal crônica, reoperação, número de procedimentos combinados (trocas ou plastias valvares múltiplas), tipo de cirurgia e o uso de concentrado de hemácias e hemocomponentes.

A escala proposta, considerando as principais variáveis do modelo múltiplo, é plausível e, quando combinada com outras variáveis dependentes, pode auxiliar na predição do risco de sangramento pós-operatório, permitindo a adoção de medidas clínicas antecipadas e precisas.

### Limitações

Este trabalho constitui um estudo unicêntrico, que por ser retrospectivo e baseado em banco de dados, não teve uma população adequada. Além disso não houve validação externa, porém esta está prevista para ser realizada num trabalho posteriormente.
