# Supplementary material for: Fatores Preditores de Risco de Sangramento em Pacientes Submetidos a Cirurgia Valvar
Source: Arq Bras Cardiol. 2024 Oct 8;121(10):e20230453. [Article in Portuguese] doi: 10.36660/abc.20230453 (PMC11634210; doi:10.36660/abc.20230453)
Supplement: Translation

# Translation: Predictive Factors for Bleeding Risk in Patients Undergoing Valvular Surgery

**DOI:** 10.36660/abc.20230453i

**Authors:** Alef de Carvalho Vieira, Renato Tambellini Arnoni, Ana Beatriz Silva Barbosa, Attila Santos Berriel, Rafael Guimarães Vianna, Mario Issa

**Affiliations:** 1 São Paulo SP Brazil Instituto Dante Pazzanese de Cardiologia, São Paulo, SP – Brazil; 2 São Paulo SP Brazil Hospital Edmundo Vasconcelos, São Paulo, SP – Brazil

**Keywords:** Valvular heart disease, Cardiovascular Surgery, Postoperative Bleeding, Risk score

## Abstract

**Background:**

The postoperative period of heart valve surgery is challenging due to the risk of bleeding, leading to complications and increased morbidity and mortality.

**Objective:**

To develop a risk score to predict bleeding in patients after valve surgery.

**Methods:**

Retrospective study of patients operated on between 2021 and 2022. Patients with major bleeding were selected based on the BARC and Bojar criteria. A logistic regression analysis was performed for factors related to bleeding and a nomogram of scores was created. For statistical significance, p<0.05 and a 95% confidence interval were considered. The study was approved by the CEP.

**Results:**

525 patients were analyzed, with a mean age of 56 years and a predominance of females. The most common valve disease was mitral insufficiency, 8.8% had increased bleeding and 4.3% had surgical reoperations. The variables with statistical significance were tricuspid insufficiency (OR 3.31, p < 0.001), chronic kidney disease/acute kidney injury (OR 2.97, p = 0.006), preoperative hemoglobin (OR 0.73, p < 0.001), reoperations (OR 2, 5, p = 0.003), cardiopulmonary bypass (CPB) time (OR 1.12, p < 0.001), 2-valve approach OR of 2.23 (p = 0.013), use of packed red blood cells OR of 2.8 (p = 0.001). In the multiple model, tricuspid insufficiency, CPB time and preoperative hemoglobin reached statistical significance.

**Conclusion:**

CPB time, preoperative hemoglobin and tricuspid insufficiency were independently associated with postoperative bleeding. The proposed scale is plausible and can help predict the risk of bleeding.

## Introduction

Cardiovascular diseases (CVDs) constitute one of the primary causes of mortality worldwide. Factors such as population aging, obesity, sedentary behavior, and chronic illnesses contribute significantly to the escalating prevalence of CVDs.^1,2^

The human heart is constituted of four valves: the mitral, aortic, tricuspid, and pulmonary valves. Conditions affecting these structures represent a significant share of CVDs and make for one-third of all cardiovascular surgery performed annually in Brazil.^3^

Surgical treatment of heart valve diseases presents its challenges, the post-operative management is a crucial one. A broad spectrum of complications can arise in this period and excessive bleeding remains a primary concern, occurring in approximately 20% of cases.^4,5^ The use of cardiopulmonary bypass,^6–9^ hypothermia, exacerbated inflammatory response and reperfusion injury can contribute to disturbances of the coagulation cascade.^6,10^

Patients submitted to reoperations face a greater risk of complications due to the necessity of detachment of adherences, friable tissues, and longer cardiopulmonary bypass time. These combined factors contribute to increased perioperative bleeding.^4,8,10,11^

The defining criteria of postoperative major bleeding varies between authors.^5,12^ The Bleeding Academic Research Consortium (BARC) has established specific criteria for grading hemorrhage, ranging from 0 to 5, in which zero indicates absence of bleeding, and five represents a fatal one (Figure 1).^13^ Bojar^14^ presents more detailed criteria, that evaluates bleeding on an hourly basis during the early postoperative period (Table 1). Kouchoukos et al.^15^ provide recommendations for a surgical hemostasis review, based on blood volume drained per hour and/or massive sudden drainage of blood.^4^

**Figure 1:** *Bleeding Academic Research Consortium* (BARC) definition.^13^

**Table 1:** Clinical diagnosis of major bleeding according to Bojar^14^

**1.**	1.5 ml/kg/h per six consecutive hours (or > 100-150ml/h);
**2.**	2.0 ml/kg/h per three consecutive hours (or > 150-200 ml/h);
**3.**	3.0 ml/kg/h per two consecutive hours (or > 250-300 ml/h).

Adapted from Bojar.^14^

The use of blood derivatives is a valid management strategy for major bleeding in the postoperative period. It is estimated that 20% of all hemocomponents transfusions take place in the surgical setting.^16^ Although blood transfusion is not absent of risk to the patient,^8,9,11,16–18^ as it increases the incidence of infection, pulmonary injuries, ischemic injuries, multi-organ dysfunction, and an overall increase in morbidity and mortality.^19–22^ This study aims to develop a risk-scoring system to identify patients with a higher probability of major bleeding in the postoperative period of surgical heart valve procedures and evaluate associated risk factors.

## Methods

### Study Design

The present study is a cross-sectional, retrospective, analytical investigation of patients submitted to surgical treatment of heart valve diseases in a cardiology reference center, intending to evaluate risk factors associated with postoperative bleeding in this population.

The population is constituted of patients submitted to heart valve surgery between January 2021 and December 2022. The immediate postoperative was defined as the first 12 hours after ICU admission, and early postoperative was defined as the period up to 48 hours.

### Inclusion and exclusion criteria

Inclusion criteria were patients with acquired or congenital valve diseases; reoperations, combined procedures involving more than one valve; and patients aged equal to or above 18 years.

Exclusion criteria were combined valve surgery with coronary artery bypass graft or aorta, patients with incomplete information from the database, and those who refused to sign the informed consent form or were not recorded in the database.

### Variables

Predictors of increased bleeding risk were defined as variables, according to the literature. The preoperative variables considered were age, weight, height, body surface area, gender, reoperation, systemic hypertension, coagulopathy and thrombocytopenia, diabetes mellitus, chronic kidney disease or acute kidney injury, use of anticoagulants or antiplatelets drugs, serum hemoglobin, and creatine levels. Intraoperative variables were total surgery, cardiopulmonary bypass and aortic cross-clamp durations, temperature during CBP, nature of the procedure (combined or not type of surgery), heparin dosage, activated clotting time (ACT) after protamine sulfate infusion, and the use of hemocomponents and autotransfusion system. Postoperative variables included the necessity of surgical hemostasis review, volume of blood drained hourly through a mediastinal drain in the first 6 hours after ICU admission, cumulative volume in the first 12, 24, and 48 hours; occurrence of intracranial hemorrhage, use of blood derivatives and their quantity, administration of antifibrinolytics, presence of metabolic acidosis, death, and death due to bleeding.

### Data extraction

The data were systematically tabulated using the RedCap (Research Electronic Data Capture) software,^23^ utilizing the database from the institute's valvular heart disease sector. An analysis of the risk factors was conducted to identify those most significantly associated with increased postoperative bleeding. The BARC^13^ and Bojar^14^ classifications were applied to differentiate between groups. For the BARC^13^ classification, criteria from item 1 were used to indicate the absence of bleeding, while item 04 from Figure 1 was used to identify cases of increased bleeding. This includes reoperation for hemostasis revision, intracranial hemorrhage within 48 hours, and blood drainage of 2000ml within the first 24 hours. In the Bojar classification,^14^ patients were assessed based on the volume of blood drained at each postoperative time interval, as detailed in Table 1.

### Statistical analysis

The sample description was performed using absolute and relative frequency measures for categorical variables, and central tendency measures (mean and median) as well as dispersion measures (standard deviation, interquartile ranges) for continuous variables. The impact of demographic, intraoperative, and surgical variables on major bleeding was assessed using logistic regression models. Initially, univariate analyses were conducted. Variables that demonstrated statistical significance were subsequently included in the multivariate analysis, considering collinearity among variables, statistical significance, and theoretical plausibility for inclusion in the model. Statistical significance was determined at a threshold of p < 0.05, with a 95% confidence interval.

Based on the final model, a nomogram was developed, using the R software. This nomogram features a series of scales that represent patient characteristics (variables included in the multivariate analysis). Each scale assigns a score, which, when summed, yields a final score for each patient. This final score is utilized to evaluate the patient's risk of bleeding.

External validation using an alternative database is not within the scope of this study. The analyses were performed utilizing the R software, version 4.2.1.

### Ethical considerations

The project was approved by the Research Ethics Committee of the Dante Pazzanese Institute of Cardiology. Number of CAAE: 59774422.9.0000.5462. Opinion number: 5.497.323. CEP number: 5268. The Informed Consent Form was waived by the committee.

## Results

### Sample description

From a total of 590 patients submitted to surgical treatment of valvular heart disease, 65 were excluded, remaining a cohort of 525 patients to be included in the analyses. The mean age of the population was 56 years old, with 57.3% being female. The average weight was 73 kg and the mean body surface area was 1.82 m². The most prevalent valve pathology was mitral regurgitation (51.2%), followed by mitral (37.7%) and aortic (33.9%) stenosis, and tricuspid regurgitation present in 19.6% of all cases. Reoperations represented 30.5% of total procedures.

Systemic arterial hypertension had a prevalence of 51%, while 16% of patients were diabetics, 9.7% had either chronic kidney disease or acute kidney injury and 18.7% had thrombocytopenia before surgery. Mean serum creatine and hemoglobin were 1.11g/dL and 12.87 g/dL, respectively, average activated partial thromboplastin time was 28.04 seconds, with a mean fibrinogen of 352 mg/dL. Out of the 525 patients, 5.5% used direct oral anticoagulants, and 24.6% used warfarin in the preoperative period.

The average surgery duration was 309.5 minutes, 123.8, and 86.91 minutes of CBP and aortic cross-clamp time, respectively. The mitral valve was addressed in 67.4% of all procedures, the aortic valve in 43.9%, and the tricuspid and pulmonary valves were involved in 15.3% and 0.6% of surgeries. During the intraoperative period, 42.3% of patients received red blood cell concentrate (RBC), with a mean quantity of 1.94 units. Blood derivatives transfusion was necessary for 31.6% of procedures, and 33.3% required the use of an autotransfusion system.

Intracranial hemorrhage in the first 48 hours had an occurrence of 0.2%, and 4.8% of patients required a surgical approach to the increased bleeding, being a thoracic diffuse unspecific hemorrhage as the cause. Patients presented a cumulative mean drained blood volume of 257 ml in 12 hours, 380 ml in 24 hours, and 475 ml in 48 hours. In the postoperative period, around 11.8% of patients required RBC concentrate transfusion, averaging 2.56 units of RBC concentrate per patient. With an overall mortality rate of 8.8%, 10.9% of those were attributable to bleeding.

### Variables related to increased bleeding

Using the BARC definition, a prevalence of major bleeding was identified as 5.3% (n=28), whereas the Bojar classification identified 40 cases (7.6%). A total of 22 patients met both the Bojar and BARC criteria to qualify as postoperative increased bleeding. The overall prevalence according to both definitions was 8.8% (n=46), with the BARC classification accounting for 61% of patients, and the Bojar classification accounting for 87%. Table 2 summarizes the variables according to the presence of major bleeding.

**Table 2:** Variables related to major bleeding

Variable		Major bleeding	
		No, N = 479	Yes, N = 46
**BARC**			
	No Major Bleeding	479 / 479 (100%)	18 / 46 (39%)
	Major Bleeding	0 / 479 (0%)	28 / 46 (61%)
**Bojar**			
	No Major Bleeding	479 / 479 (100%)	6 / 46 (13%)
	Major Bleeding	0 / 479 (0%)	40 / 46 (87%)
**Bleeding Volume (1^st^ hour)**		25 (0. 50)	212 (100. 344)
**Bleeding Volume (48 hours)**		400 (250. 525)	1.200 (750. 1.475)
**Bleeding (BARC)**			
	No major bleeding	479 / 479 (100%)	18 / 46 (39%)
	Type 4	0 / 479 (0%)	1 / 46 (2.2%)
	Type 4a	0 / 479 (0%)	1 / 46 (2.2%)
	Type 4b	0 / 479 (0%)	24 / 46 (52%)
	Type 4c	0 / 479 (0%)	1 / 46 (2.2%)
	Type 5b	0 / 479 (0%)	1 / 46 (2.2%)
**Intracranial hemorrhage (up to 48h)**			
	No	479 / 479 (100%)	45 / 46 (98%)
	Yes	0 / 479 (0%)	1 / 46 (2.2%)
**Reoperation due to major bleeding**			
	No	479 / 479 (100%)	22 / 46 (48%)
	Yes	0 / 479 (0.0%)	24 / 46 (52%)
**RBC Concentrate (up to 48h)**			
	No	447 / 479 (93%)	16 / 46 (35%)
	Yes	32 / 479 (6.7%)	30 / 46 (65%)
**Blood Components Transfusion (up to 48h)**			
	No	470 / 479 (98%)	16 / 46 (35%)
	Yes	9 / 479 (1.9%)	30 / 46 (65%)
**Quantity of Platelets Concentrates**		6.0 (5.0. 7.8)	8.0 (6.0. 10.0)
**Imediate Postoperative Metabolic Acidosis**			
	No	304 / 479 (63%)	22 / 46 (48%)
	Yes	175 / 479 (37%)	24 / 46 (52%)
**Death**			
	No	447 / 479 (93%)	32 / 46 (70%)
	Yes	32 / 479 (6.7%)	14 / 46 (30%)
**Death Secondary to Bleeding**			
	No	32 / 32 (100%)	9 / 14 (64%)
	Yes	0 / 32 (0%)	5 / 14 (36%)

RBC: red blood cell.

### Univariate analysis according to the preoperative period

Among the preoperative variables analyzed, those that achieved statistical significance were tricuspid insufficiency with an odds ratio (OR) of 3.31 (95% confidence interval [CI] 1.74 - 6.20, p < 0.001), the presence of chronic kidney disease or acute kidney injury with an OR of 2.97 (95% CI 1.31 - 6.23, p = 0.006), preoperative hemoglobin with an OR of 0.73 (95% CI 0.62 - 0.85, p < 0.001), and reoperation with an OR of 2.5 (95% CI 1.35 - 4.62, p = 0.003).

The analyzed surgical variables included the median duration of the procedure, which was 327.5 minutes, with an OR of 1.05 (95% CI 1.03 - 1.10, p < 0.001); CBP time of 130 minutes, OR 1.12 (95% CI 1.06 - 1.18, p < 0.001); aortic cross-clamp of 90 minutes, OR 1.13 (95% CI 1.05 - 1.21, p < 0.001. The effects were evaluated considering 10-minute increments for each variable. For example, each additional 10 minutes of cardiopulmonary bypass is associated with a 12% increase in the likelihood of major bleeding.

The involvement of two valves requiring repair/replacement during the surgical procedure was significantly associated with an increased risk of bleeding, with an OR of 2.34 (95% CI 1.17 - 4.53, p = 0.013). A triple valve approach showed a heightened risk with an OR of 3.7 (95% CI 1.00 - 11.1, p – 0.028). Additionally, different valve combinations demonstrated significant risk increases, such as mitral valve replacement and tricuspid valve repair, OR 3.4 (95% CI 1.33 - 8.66, p = 0.01), and the combined approach of mitral, aortic, and tricuspid interventions, OR 3,79 (95% CI 0.96 0 12.8, p = 0.04).

RBC concentrate transfusion demonstrated a statistically significant association with major bleeding, with an OR of 2.8 (95% CI 1.51 - 5.4, p = 0.001). Additionally, the administration of two or more units increased the likelihood of bleeding, OR 1.52 (CI 95% 1.06 - 2.16, p = 0.021). Furthermore, the use of an autotransfusion system was also associated with a higher risk of bleeding, OR 2.61 (95% CI 1.42 - 4.85, p = 0.002).

### Multivariate analysis

The variables that retained statistical significance in the multivariate model were preoperative hemoglobin, cardiopulmonary bypass time, and the presence of tricuspid regurgitation, as detailed in Table 3. Tables 4 and 5 evaluate other relevant variables, such as chronic kidney disease, reoperation, and the use of RBC concentrate. Despite their clinical relevance, these variables did not achieve statistical significance in the multivariate analysis.

**Table 3:** Multivariate analysis considering preoperative hemoglobin, CPB time, and tricuspid regurgitation

Variable		OR	95% IC	p Value
Preoperative Hemoglobin		0.76	0.64; 0.89	0.001
CPB Time (each additional 10 minutes)		1.09	1.03; 1.15	0.004
**Tricuspid Regurgitation**				
	No	—	—	
	Yes	2.04	1.01; 4.06	0.043

CPB: cardiopulmonary bypass.

**Table 4:** Multivariate analysis considering preoperative hemoglobin, CPB time, tricuspid regurgitation, red blood cell concentrate transfusion, and reoperation

Variable		OR^1^	95% IC	p Value
**Intraoperative RBC transfusion**				
	No	—	—	
	Yes	1.17	0.54; 2.57	0.7
**Reoperation**				
	No	—	—	
	Yes	1.34	0.65; 2.72	0.4
Preoperative Hemoglobin		0.79	0.65; 0.95	0.012
CPB Time/10		1.08	1.01; 1.14	0.016
**Tricuspid regurgitation**			
	No	—	—	
	Yes	2.01	0.99; 3.99	0.049

CPB: cardiopulmonary bypass.

**Table 5:** Multivariate analysis considering preoperative hemoglobin, chronic kidney failure or acute kidney injury, CPB time, and reoperation

Variable		OR	95% CI	p Value
Preoperative Hemoglobin		0.79	0.66; 0.94	0.009
CPB Time /10		1.09	1.03; 1.16	0.003
**Previous kidney lesion (Chronic/acute)**				
	No	—	—	
	Yes	2.14	0.86; 4.97	0.087
**Reoperation**				
	No	—	—	
	Yes	1.54	0.75; 3.11	0.2

CPB: cardiopulmonary bypass.

Figures 2 and 3 present the nomograms related to the analyses shown in Tables 3 and 5, which provide a scoring system to estimate the risk of postoperative bleeding.

**Figure 2:** Bleeding risk nomogram related to the model presented in Table 3.

**Figure 3:** Bleeding risk nomogram related to the model presented in Table 5.

## Discussion

Postoperative increased bleeding lacks a uniform definition, which renders its identification and management more complex. It involves a wide variety of factors that contribute to its presentation and lead to an increase in morbidity and mortality.^4,24,25^

This study aimed to emphasize the characteristics of patients undergoing heart valve surgery and to examine their association with increased postoperative bleeding. The population had a mean age of 56 years and 57.3% were female. While these findings are consistent with previous studies, it is crucial to underscore the correlation between age over 75 years, body mass index below 25 kg/m², and reduced body surface area as significant risk factors for bleeding.^4,26–29^

Mitral regurgitation was identified as the most prevalent valvular pathology in this cohort (51.2%). Previous studies, such as those by Vuylsteke et al.,^29^ have highlighted a significant correlation between aortic stenosis and the risk of bleeding, attributed to the impairment of the Von Willebrand factor and other components of the coagulation cascade. Additionally, mitral valve disease is often associated with atrial fibrillation and the use of anticoagulants such as warfarin, which may contribute to postoperative bleeding.^29^

Approximately 8.8% of patients experienced increased bleeding, with an average drainage of 380 ml at 24 hours and 475 ml at 48 hours. Moreover, 4.3% required reintervention for hemostasis control. The findings are consistent with previous literature, where the rates ranged from 6.4% to 12%, and from 2.4% to 9.5% for bleedings and reinterventions, respectively. However, other studies reported higher bleeding volumes, with drainage ranging from 525 ml to 1669 ml at 24 hours.^4,10,12,24–26,28,29^

According to Vuylsteke et al.,^29^ definitions that consider bleeding rated concerning hour and weight are more reliable. Nonetheless, overall accounting may not be accurate, as intraoperative bleeding is not included, and potential human errors in reporting can lead to inaccurate volume estimations. In addition, using the number of blood derivative units as a marker for defining major bleeding may lack precision, as the decision to transfuse is subject to the judgment of the attending physician.^29^

Patients with chronic kidney disease or acute kidney injury exhibit poorer mortality outcomes in the context of valvular heart disease.^30^ Furthermore, this study identified an association with increased bleeding, which can be attributed to reductions in both platelet quantity and quality, decreased levels of coagulation factors, and chronic anemia – each contributing to compromised coagulation function.^30^

A positive association was observed between preoperative hemoglobin levels and postoperative bleeding. The findings of this study align with reports by Bailly et al.,^28^ which was 13.6 g/dL. This study demonstrated that each 1g/dL increase in hemoglobin levels was associated with a 27% reduction in the risk of bleeding. This effect is likely due to higher hemoglobin levels improving the transport of energetic substrates, which enhances platelet function and coagulation cascade activity and reduces the likelihood of requiring RBC transfusions during surgery.

Blood transfusion may be required in up to 21% of cases during surgery and up to 5% in the postoperative period, as reported in previous studies.^28^ In contrast, the present study observed rates of 42.3% during the procedure and 2.56% postoperatively. Furthermore, the administration of two or more units of RBC concentrate was associated with a 1.52-fold increase in the risk of bleeding. This finding is particularly noteworthy as excessive RBC transfusion is linked to increased morbidity and mortality in the postoperative period.

It was observed that postoperative bleeding in patients undergoing reoperation is 2.5 times greater, which is consistent with findings from other studies in the field. These patients typically experience longer surgical and CPB times due to the more delicate and complex surgical techniques required.^26^

The duration of CPB is closely linked to increased rates of postoperative bleeding. CPB time plays a crucial role, affecting other temporal factors such as surgical duration and aortic cross-clamping, as well as contributing to additional factors that heighten the risk of bleeding. Extended CPB times lead to greater consumption of coagulation factors, cellular damage, increased fibrinolysis, and hemodilution, all of which compromise hemostatic function.^4,25^ A CPB time exceeding 150 minutes has a significant impact on postoperative bleeding. In this study, each additional 10 minutes of CPB was associated with a 12% increased risk of bleeding.^4,26,30^

There is an increased risk of bleeding as additional valves are addressed for replacement or repair, with the risk rising by up to 2 to 3.7 times. The association is clearly influenced by the duration of CPB, as additional time is required to address each valve.^26,29,30^

In the multivariate analysis, tricuspid regurgitation, duration of CPB, and preoperative hemoglobin levels were identified as the primary factors associated with bleeding. This finding contrasts with previous literature, which has predominantly associated aortic stenosis with bleeding complications. The reasons for this discrepancy are not yet fully understood but may be related to the differing incidence of tricuspid regurgitation and variations in surgical approaches when multiple valvular pathologies are present.^4,12,24,26,29^

Several variables, including the use of RBC concentrates, reoperations, and chronic kidney disease, significantly influence postoperative bleeding rates, although they do not do so independently. Notably, while chronic kidney disease is recognized for its pathophysiological role in increasing bleeding risk, this study did not find an independent association with bleeding outcomes. Furthermore, potential confounding factors were not evaluated, which may affect the interpretation of these findings.^30^

Previous research has identified several additional significant risk factors that should be considered, including advanced age, non-elective surgeries, and the dosage of heparin and protamine administered during the procedure.^4,12,26,29^

Therefore, the development of the nomogram may be useful for estimating a patient's bleeding risk. However, external validation of this scoring system through further studies is necessary.

### Limitations

This study is a single-center, retrospective analysis based on a database, which may limit the representativeness of the population. Additionally, external validation was not conducted, though plans for such validation are anticipated in future research.

## Conclusion

Based on the analyses conducted, it is concluded that cardiopulmonary bypass time, the presence of tricuspid regurgitation, and preoperative hemoglobin levels are independently associated with postoperative bleeding.

Furthermore, additional variables have shown significant associations with postoperative bleeding. These include chronic kidney disease, reoperation, the number of combined procedures (such as a multiple valve approach), the type of surgery performed, and the use of RBC concentrate and blood derivatives.

The proposed scale, which integrates the primary variables from the multivariate model, demonstrates theoretical plausibility. When used in conjunction with other dependent variables, it has the potential to enhance the prediction of postoperative bleeding risk. This could facilitate the adoption of timely and precise clinical interventions.
